# Neurophysiological signatures of hand motor response to dual-transcranial direct current stimulation in subacute stroke: a TMS and MEG study

**DOI:** 10.1186/s12984-020-00706-1

**Published:** 2020-06-11

**Authors:** I-Ju Kuo, Chih-Wei Tang, Yun-An Tsai, Shuen-Chang Tang, Chun-Jen Lin, Shih-Pin Hsu, Wei-Kuang Liang, Chi-Hung Juan, Catharina Zich, Charlotte J. Stagg, I-Hui Lee

**Affiliations:** 1 Institute of Brain Science, Brain Research Center, National Yang-Ming University, No.155, Sec. 2, Linong St., Beitou Dist, Taipei City, 112 Taiwan; 2 Department of Neurosurgery, Taipei Veterans General Hospital, No.201, Sec. 2, Shipai Rd., Beitou Dist, Taipei City, 112 Taiwan; 3 Department of Neurology, Far Eastern Memorial Hospital, No.21, Sec. 2, Nanya S. Rd., Banqiao Dist, New Taipei City, 220 Taiwan; 4 Division of Cerebrovascular Diseases, Neurological Institute, Taipei Veterans General Hospital, No.201, Sec. 2, Shipai Rd., Beitou Dist, Taipei City, 112 Taiwan; 5 Institute of Cognitive Neuroscience, National Central University, No.300, Zhongda Rd., Zhongli Dist, Taoyuan City, 320 Taiwan; 6 Wellcome Centre for Integrative Neuroimaging, FMRIB, Nuffield Department of Clinical Neurosciences, University of Oxford, Oxford, OX3 9DU UK; 7 Oxford Centre for Human Brain Activity, Wellcome Centre for Integrative Neuroimaging, Department of Psychiatry, University of Oxford, Oxford, OX3 7JX UK; 8 MRC Brain Network Dynamics Unit, University of Oxford, Oxford, OX1 3TH UK

**Keywords:** Subacute stroke, Transcranial direct current stimulation, Transcranial magnetic stimulation, Transcallosal inhibition, Magnetoencephalography, Plasticity

## Abstract

**Background:**

Dual transcranial direct current stimulation (tDCS) to the bilateral primary motor cortices (M1s) has potential benefits in chronic stroke, but its effects in subacute stroke, when behavioural effects might be expected to be greater, have been relatively unexplored. Here, we examined the neurophysiological effects and the factors influencing responsiveness of dual-tDCS in subacute stroke survivors.

**Methods:**

We conducted a randomized sham-controlled crossover study in 18 survivors with first-ever, unilateral subcortical ischaemic stroke 2–4 weeks after stroke onset and 14 matched healthy controls. Participants had real dual-tDCS (with an ipsilesional [right for controls] M1 anode and a contralesional M1 [left for controls] cathode; 2 mA for 20mins) and sham dual-tDCS on separate days, with concurrent paretic [left for controls] hand exercise. Using transcranial magnetic stimulation (TMS) and magnetoencephalography (MEG), we recorded motor evoked potentials (MEPs), the ipsilateral silent period (iSP), short-interval intracortical inhibition, and finger movement-related cortical oscillations before and immediately after tDCS.

**Results:**

Stroke survivors had decreased excitability in ipsilesional M1 with a relatively excessive transcallosal inhibition from the contralesional to ipsilesional hemisphere at baseline compared with controls, as quantified by decreased MEPs and increased iSP duration. Dual-tDCS led to increased MEPs and decreased iSP duration in ipsilesional M1. The magnitude of the tDCS-induced MEP increase in stroke survivors was predicted by baseline contralesional-to-ipsilesional transcallosal inhibition (iSP) ratio. Baseline post-movement synchronization in α-band activity in ipsilesional M1 was decreased after stroke compared with controls, and its tDCS-induced increase correlated with upper limb score in stroke survivors. No significant adverse effects were observed during or after dual-tDCS.

**Conclusions:**

Task-concurrent dual-tDCS in subacute stroke can safely and effectively modulate bilateral M1 excitability and inter-hemispheric imbalance and also movement-related α-activity.

## Background

Transcranial direct current stimulation (tDCS) has been demonstrated to non-invasively modulate cortical excitability in the primary motor cortex (M1) in both controls and stroke survivors [1, 2]. Motor evoked potentials (MEPs) typically increase following unilateral anodal tDCS and decrease following unilateral cathodal tDCS, with effects outlasting stimulation by minutes to hours [3]. This polarity-specific modulation has been suggested as a putative way to promote post-stroke motor recovery, either by enhancing ipsilesional M1 excitability with anodal tDCS or decreasing contralesional M1 excitability with cathodal tDCS [4]. Some tDCS studies have suggested that these approaches may be promising, but the quality of evidence for tDCS in promoting post-stroke motor recovery is still low to moderate [5], partially due to heterogeneity in study designs and stroke survivor profiles across the literature, and small sample sizes within studies [6]. To successfully translate tDCS effects into clinical benefits, patient selection based on neurophysiological status for a given effective tDCS montage may be required [6–9].

Dual, or bi-hemispheric, tDCS involves concurrent anodal stimulation to one M1 and cathodal stimulation to the other M1. The effects of dual tDCS have been studied in both healthy controls and stroke survivors in a number of different ways. Dual-tDCS has been shown to have a significant beneficial effect in dexterity [10] and motor learning [11–14] in controls. The effects of dual-tDCS on motor performance were better or at least equal to the unilateral anodal stimulation effect in most of these studies [10, 11, 13, 14]. Consistent with these behavioural findings, increased MEPs in the anode-targeted M1 and decreased MEPs in the cathode-targeted M1 after dual-tDCS have been demonstrated in most studies in healthy controls [10, 14–17], suggesting that dual-tDCS may result in additive effects of unilateral stimulation, although these effects are not entirely consistent [18].

The effects of dual-tDCS post stroke, when an ipsilesional anode and a contralesional cathode have been paired with concurrent rehabilitation, are much more varied across studies, with some studies demonstrating significantly enhanced motor score, dexterity or grip strength [19–23] in chronic stroke survivors, but other studies showing no effect [24–26]. Only one previous study has explored the immediate post-stimulation effects of dual-tDCS on cortical excitability, which showed no significant changes in MEPs or transcallosal inhibition in six subacute stroke survivors [27]. Two studies have combined repetitive dual-tDCS with rehabilitation in chronic stroke survivors and demonstrated an increase in ipsilesional MEPs compared to sham [19, 20]. Sensorimotor dynamic activity, recorded using electroencephalography (EEG) or magnetoencephalography (MEG), are also altered following stroke [28–31], and changes in these measures are correlated with motor function [30, 31]. The event-related desynchronization (ERD) before and during movement has been associated with motor preparation and execution, while event-related synchronization (ERS) after cessation of movement has been suggested to reflect motor deactivation [32]. Dual-tDCS has been shown to alter motor imagery-related hemispheric lateralization in sensorimotor rhythms in controls [33] and after stroke [34]. However, the effect of dual-tDCS on movement-related dynamic activity has not yet been examined.

We hypothesized that task-concurrent dual-tDCS could enhance ipsilesional corticospinal excitability, modulate movement-related brain oscillations, and also rebalance the asymmetry of interhemispheric interactions after subacute stroke. Potential for recovery is most prominent early after stroke and declines gradually after 6 months (chronic stage) [35, 36]. Most rehabilitation intervention studies, including tDCS studies, recruit patients at the chronic stage of recovery [37], but it is likely that rehabilitative effects would be stronger if applied earlier. In addition, recent studies have also demonstrated that different adaptive mechanisms and bi-hemispheric interactions may exist during the subacute and chronic stages [38, 39]. Studying effects in subacute stroke is therefore important if we are to fully explore the potential of dual-tDCS for stroke recovery. Cortical and subcortical infarctions have highly different outcomes, network reorganization and tDCS responsiveness [22]. To minimize variations in tDCS substrates (the cortex) and responsiveness in this study, we enrolled stroke survivors with relatively homogenous subcortical infarctions at 2–4 weeks post-stroke to investigate the effects of dual-tDCS using transcranial magnetic stimulation (TMS) and MEG in a randomized, sham-controlled, crossover study design to compare the effects of dual-tDCS in subacute stroke survivors and age- and gender-matched controls, as well as explore factors predicting responsiveness to dual-tDCS.

## Materials and methods

### Subjects and study design

We recruited patients aged 20–80 years with first-ever, unilateral, ischaemic stroke with mild to moderate hand paresis (Medical Research Council motor score 3–4) 2–4 weeks after stroke. Exclusion criteria were: major neurological or medical comorbidities or absent MEPs in the paretic hand. Age- and gender-matched healthy controls were also recruited. All participants gave their informed consent to participate in the study, which was approved by the institutional review board of Taipei Veterans General Hospital.

**Fig. 1:**
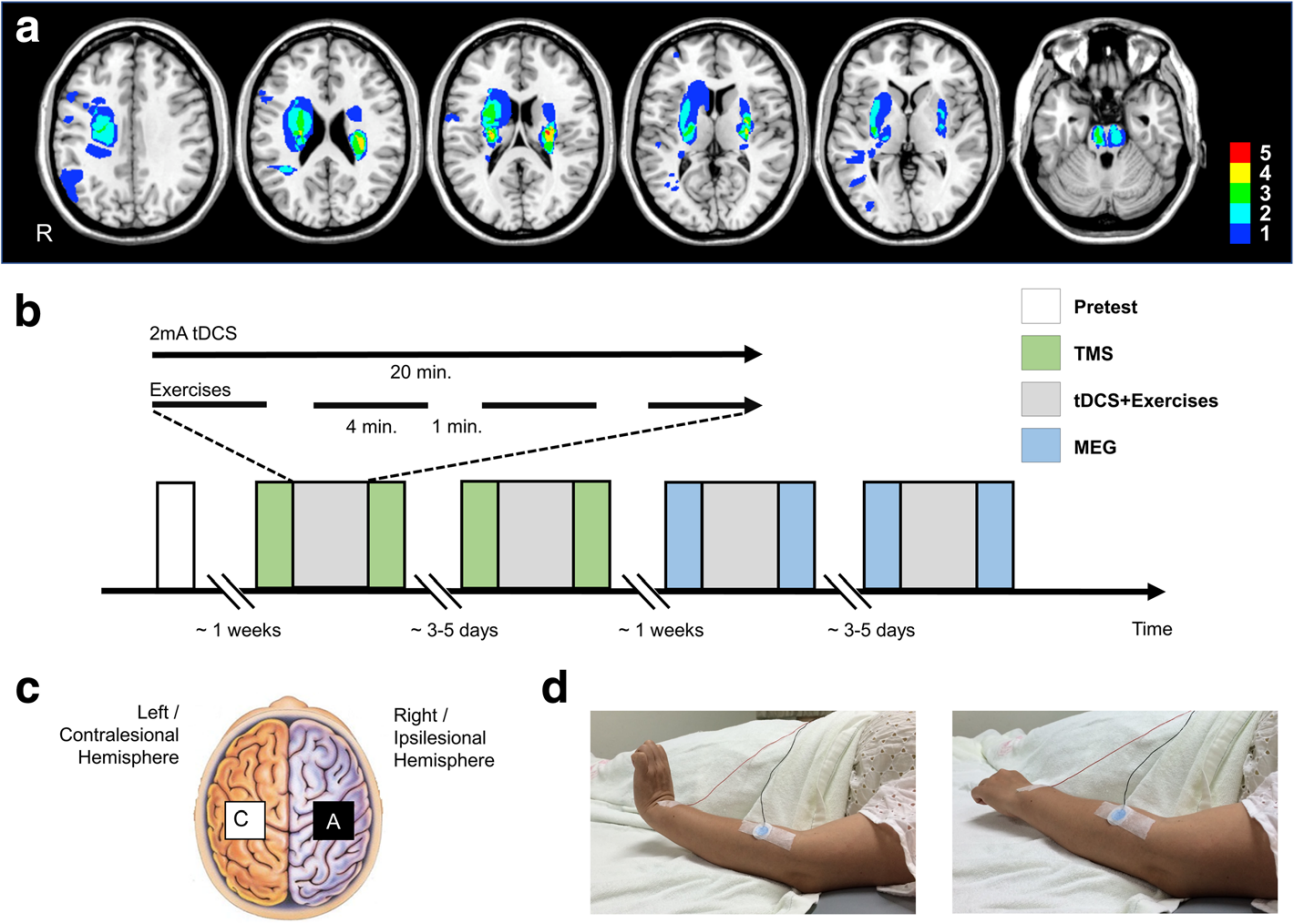
Stroke lesion map and study design. **a** The overlapped lesion map of the stroke survivors (*N* = 18). The colour spectrum represents the number of patients containing lesions at the corresponding locations. **b** The crossover study design. Four dual-transcranial direct current stimulation (tDCS) sessions were performed (TMS + real tDCS, TMS + sham tDCS, MEG + real tDCS, MEG + sham tDCS) for each participant. The order of the sessions was counterbalanced across the groups. **c** Schematic illustration of dual-tDCS montage, with anodal electrode over right or ipsilesional hemispheric primary motor cortex (M1) and cathodal electrode over left or contralesional M1. **d** picture illustrating the wrist extension movement performed during tDCS stimulation or TMS measurements with surface EMG monitor. See Methods for details TMS = transcranial magnetic stimulation; MEG = magnetoencephalography; EMG = electromyography

Twenty-one stroke survivors fulfilled the inclusion/exclusion criteria and 3 of them dropped out (14%) after recruitment because of unwillingness to complete the full assessment. The remaining 18 stroke survivors were enrolled a median of 23 days (range:14–28) after stroke. Survivors had a median age of 63 (range:31–76 years) and equal gender distribution (Table 1). They had mild to moderate hand paresis as defined by the Medical Research Council (MRC) score 3–4 of the extensor carpi radialis (ECR) muscle for being suitable for TMS measures from the ECR and MEG measures of finger lifting task. Their median scores of Fugl-Meyer assessment-upper extremity scale (FMA-UE) and Action Research Arm Test (ARAT) were 60 (range:24–64) and 56 (range:3–57), respectively. All stroke survivors were right-handed [40], and 10 of them had left hemiparesis. Lesions were mainly located in the deep nuclei and white matter of the middle cerebral artery territory. Four subjects had a pons infarction (Fig. 1a). 14 age (median:59, range:37–72) and gender (6 male) matched right-handed controls were also enrolled [Age: U = 78, *p* = 0.07; Gender χ^2^(1) = 0.69, *p* = 0.74].

**Table 1:** Baseline Characteristics of Subacute Stroke Survivors

No/lesion	Age/Sex	Post-stroke (d)	NIHSS 2-4w	mRS 2-4w	MRC ECR	FMA-UE	ARAT	rMT (%)	MEP (μV)	iSP (ms)	SICI (ratio)
1 /L	67/M	28	2	4	4	63	56	50/47	85/134	62/52	0.31/0.39
2 /R	68/F	14	1	4	3	62	57	39/38	312/677	72/52	0.18/0.25
3 /L	41/F	25	6	4	3	49	24	90/47	52/1710	NA/56	0.43/0.51
4 /R	31/M	23	1	4	4	63	57	36/31	145/468	77/78	0.25/0.23
5 /R	76/M	22	1	2	4	64	57	38/38	294/628	82/77	0.43/0.46
6 /R	66/M	20	4	3	3	24	3	42/35	912/561	84/72	0.46/0.22
7 /R	75/F	15	5	4	4	43	38	63/49	244/628	69/61	0.38/0.24
8 /L	70/M	19	2	4	3	32	6	36/30	645/2196	90/67	0.11/0.32
9 /L	44/F	23	2	4	3	60	56	45/39	157/996	92/47	0.26/0.19
10/L	56/F	18	1	3	4	64	57	42/41	297/638	84/52	0.32/0.35
11/R	56/M	15	1	3	4	64	57	30/32	613/648	79/53	0.21/0.30
12/L	60/F	14	0	2	4	61	57	40/33	388/721	64/62	0.12/0.24
13/L	63/M	28	2	3	4	53	32	100/45	70/787	NA/NA	NA/0.28
14/R	62/F	26	5	3	4	61	57	52/46	101/838	74/64	0.45/0.27
15/R	66/F	28	5	4	3	28	6	46/39	160/1309	93/68	0.40/0.61
16/R	61/M	18	3	3	4	43	48	88/46	109/976	79/64	NA/0.38
17/R	76/F	28	1	1	4	60	57	51/39	346/1060	77/74	0.28/0.46
18/L	61/M	28	5	4	3	40	20	38/32	154/2069	118/64	0.22/0.27
**8 L/10R**	**63** (57–68) **9 M/9F**	**23** (18–28)	**2** (1–5)	**4** (3–4)	**4** (3–4)	**60** (43–63)	**56** (26–57)				

*NIHSS* National Institutes of Health Stroke Scale, *mRS* Modified Rankin Scale, *MRC* Medical Research Council scale of the extensor carpi radialis (ECR) muscle strength, *FMA-UE* Fugl-Meyer Assessment - Upper Extremity, *ARAT* Action Research Arm Test, *d* Days, *w* Weeks. The individual electrophysiologic measurements using transcranial magnetic stimulation were recorded from the affected/unaffected ECR, including rMT: resting motor threshold (% of maximum stimulator output); *MEP* Motor evoked potential, *iSP* Ipsilateral silent period, *SICI* Short-interval intracortical inhibition. *NA* Not accessible (recordable), *M* Male, *F* Female, *R* Right hemisphere, *L* Left hemisphere. The group values represent the median (interquartile range)

All subjects participated in four experimental sessions on separate days: two real and two sham dual-tDCS sessions, which were combined with either TMS or MEG recordings (i.e. TMS + real tDCS, TMS + sham tDCS, MEG + real tDCS, MEG + sham tDCS). The order of the sessions was counterbalanced across the groups and there was at least 72 h between sessions (Fig. 1b).

### Transcranial direct current stimulation

A tDCS stimulator (Eldith, UK) with 5x5cm conductive rubber electrodes and saline-soaked sponges was used. M1 representations were localized in each participant from ECR hotspot derived from single-pulse TMS. The anode was placed over the ipsilesional [right for controls] M1 [anode-targeted M1; M1_Anode_], and the cathode over the contralesional [left for controls] M1 [cathode-targeted M1; M1_Cathode_] (Fig. 1c). Impedance was kept below 5 kΩ. For real stimulation, a 2 mA current (current density 0.08 mA/cm^2^) was applied for 20 min, including 30 s ramp-up and ramp-down. This tDCS dosage was safe according to the safety guideline and used by previous dual-tDCS studies [6, 41]. For sham stimulation, the stimulator was turned off immediately after the initial ramp-up period.

During the tDCS, subjects performed full range self-paced extension of the paretic [left for controls] ECR, with wrist extension over 3 s followed by 3 s of wrist flexion back to neutral position. This was repeated for 4 min followed by 1 min of rest for 4 cycles (Fig. 1b and d). The Adverse Effects Questionnaire [42] was administered after each tDCS session.

### Transcranial magnetic stimulation

In the TMS sessions, MEPs, the ipsilateral silent period (iSP), and short-interval intracortical inhibition (SICI) were recorded from both M1s before (i.e. baseline) and immediately after real or sham tDCS (0, 15, 30 min) Both resting motor threshold (rMT) and active motor threshold (aMT) were measured once before either real or sham tDCS stimulation as the baseline values. The fixed evaluation order of each TMS session was rMT → MEP → SICI→iSP → aMT of the non-paretic hand [right for controls)] and then the paretic hand [left for controls]. In line with the literatures, we observed the after-effects of a single-session tDCS lasted for about 30–40 min [2]. Therefore, the TMS measurements were completed by 40 min after tDCS to be within an estimated effective window. We utilized a Magstim Rapid^2^ stimulator (Magstim, UK) with a 90 mm diameter double cone coil and a synchronized surface electromyography recording system (MedelecSynergy; VIASYS HealthCare, UK) for rMT, aMT, MEP amplitude, and iSP measurements. Two Magstim 200^2^ stimulators were used for SICI. The rMT was defined as the minimum intensity required to elicit MEPs with a peak-to-peak amplitude greater than 50 μV in 5 out of 10 trials in the relaxed ECR [43]. The aMT was defined as the minimum intensity required to evoke MEPs with a peak-to-peak amplitude greater than 200 μV in 5 out of 10 trials, while the subject maintained at ~ 20% of maximum contraction. The strength of muscle contraction was visually monitored and maintained by concurrent surface EMG recording (Fig. 1d). MEP amplitude was quantified as the mean peak-to-peak amplitude from 12 successive TMS pulses evoked every 5 s at an intensity of 120% baseline rMT. The iSP was calculated from the mean of 5 trials and was defined as the length of disrupted ongoing muscular activity of the ECR after an ipsilateral M1 TMS pulse at an intensity of 150% rMT. The participant was asked to maintain steady wrist extension with maximal strength monitored by surface EMG during the test period. The onset point of iSP was defined as disrupted ongoing muscular activity below the mean EMG amplitude of the baseline before the TMS pulse. The offset point of iSP was defined as EMG amplitude recovery to 50% of the baseline before the TMS pulse [44, 45]. The iSP ratio of contralesional / ipsilesional [left/right for controls] iSP was used to examine the interhemispheric transcallosal inhibition imbalance between contralesional to ipsilesional [left to right for controls] hemisphere. SICI was quantified using a subthreshold conditioning stimulus (80% of the baseline rMT) and suprathreshold test stimulus (120% of the baseline rMT), with individualized inter-stimulus intervals (ISI) between 1 and 5 ms to prevent ceiling or floor effects. ISI remained constant within subject across the experiment. SICI was calculated as the percentage of conditioned MEP amplitude relative to unconditioned MEPs for each trial and was obtained from the mean of 5 trials with a 5 s interval [46, 47].

### Magnetoencephalography acquisition and analysis

In the MEG sessions, movement-related cortical oscillations were recorded before (i.e. baseline), and immediately after real and sham tDCS. The MEG measurements were completed by 40 min after tDCS to be within an estimated effective window. A Neuromag Vectorview MEG (Elekta, Helsinki, Finland) was used to acquire electrophysiological data while performing a simple motor task (see [48] for more details). Briefly, subjects performed a self-paced, unilateral index finger lifting task every 7 s with the paretic [left for controls] hand, keeping other muscles relaxed and avoiding excessive blinking. The acquisition lasted for up to 15 min to collect around 50 adequate trials as determined by online MEG and electrooculography (EOG) monitoring with a pre-defined rejection threshold (4000 fT for MEG and 200 mV for EOG).

MEG data were acquired with a 500 Hz sampling rate and analysed using Brainstorm [49]. MEG epochs comprised the − 3 s to 3 s relative to movement onset, identified by an optic detection pad. Trials were discarded if the interval between two consecutive finger lifts was shorter than 6 s, or the corresponding EOG or MEG data were noisy, as defined by visual inspection and aforementioned EOG or MEG pre-defined rejection threshold. MEG channels were co-registered with the individual-MRI. MEG data were then filtered into α-band (8–12 Hz) and β-band (16–30 Hz), and projected to source level by minimum norm estimate [50]. The event-related desynchronization/synchronization (ERD/S) were then calculated as follows: ERD/ERS % = A-R/R × 100 (A: the power within the frequency band of interest during the active period of the event; R: the mean power of the reference period) [51]. The peak amplitude of ERD [or ERS] was determined as the minimum [or maximum] between − 2 to 2 s [or 0 to 3 s] relative to movement onset for each individual [51]. This procedure resulted in four MEG measures of interest, i.e. α-ERD, α-ERS, β-ERD, β-ERS peak amplitude.

### Statistical analyses

SPSS version 24.0.0 (Chicago, USA) was used for statistical analyses. Data were transformed where necessary to achieve a normal distribution. Outliers were identified if studentized residuals were greater than ±3 and removed from analysis.

Repeated measures analysis of variance (ANOVAs) were used to compare the baseline TMS (rMT, aMT, MEPs, iSP and SICI) and MEG measures (α-ERD, α-ERS, β-ERD, β-ERS) within groups (baseline session 1, baseline session 2), while Mann-Whitney U tests were used to compare these baseline TMS and MEG measures between groups (stroke survivors, healthy controls).

To investigate the effect of tDCS, TMS measures were normalized to baseline (post / baseline) and normalized values were used for all analyses of change. MEG measures were normalized as follows: (post - baseline) / (post + baseline). ANOVA with a post hoc Bonferroni correction for multiple comparisons was used to compare the normalized TMS and MEG measures. For TMS, the ISI was used as the covariate in SICI analysis.

To define tDCS responsiveness, post-tDCS MEPs changes were categorized into three groups [52]: High: if the mean at two or more time points (0, 15 mins, 30 mins) was greater than 15% for anodal tDCS or smaller than 15% for cathodal tDCS; Medium: between 10 and 15%; Low: < 10%. Stepwise multiple regression analysis with backward elimination was used to identify the independent factors for the tDCS response.

## Results

### Dual-tDCS is well tolerated

The only self-reported discomfort was tingling during the initial 1–2 min of tDCS stimulation (four subjects, 14%). No seizures or other severe adverse events were reported.

### Stroke survivors had decreased baseline excitability in ipsilesional M1 compared with controls

**Fig. 2:**
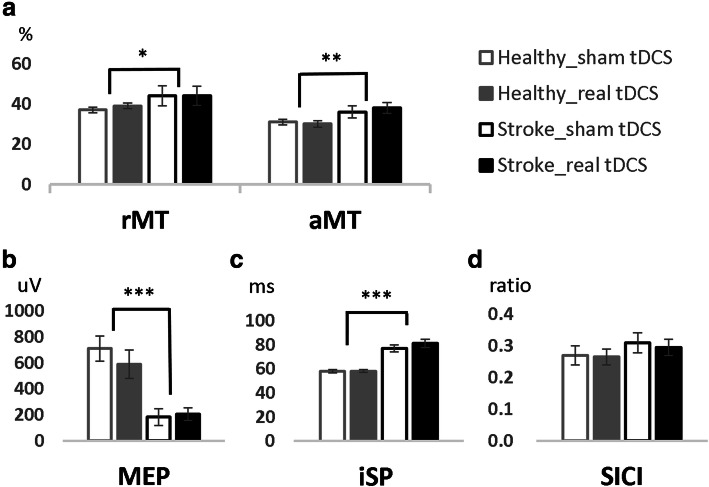
Transcranial magnetic stimulation (TMS) measurements before (baseline) real and sham tDCS. The data were recorded from paretic and non-dominant left extensor carpi radialis muscles in stroke survivors (*n* = 18) and healthy controls (*n* = 14), respectively. Mean and standard error across individuals is shown. **a** Resting and active motor threshold (rMT, aMT, % of maximum stimulator output). **b** Motor evoked potential (MEP). **c** Ipsilateral silent period (iSP). **d** Short-interval intracortical inhibition (SICI). Measures with statistical significance are indicated as: *p < 0.05, **p < 0.01, ***p < 0.001

We first wanted to investigate whether there were any differences between stroke survivors and controls in baseline TMS measures, i.e. before tDCS was applied. There were no significant intra-individual variabilities in the baseline TMS measures before real versus sham stimulation in healthy controls and stroke survivors, respectively (all *p* > 0.1, supplementary Table A.1). Therefore, we used the mean of the baseline measures for subsequent analyses. Compared to controls, stroke survivors had increased rMT (51 ± 21% versus 38 ± 5% maximum stimulator output, U = 63, *p* = 0.017, r = 0.42), increased aMT (40% ± 12% versus 30 ± 6% maximum stimulator output, U = 59, *p* = 0.009, r = 0.45), and decreased ipsilesional M1 excitability (MEP amplitude at 120% rMT 282 ± 233 μV versus 753 ± 374 μV, U = 27, *p* < 0.001, r = 0.66). Stroke survivors also showed increased contralesional-to-ipsilesional transcallosal inhibition (iSP: 81 ± 13 ms versus 58 ± 4 ms, U = 1, p < 0.001, r = 0.18), but comparable ipsilesional SICI (30 ± 12% versus 27 ± 9%, U = 122, *p* = 0.88, r = 21.6; Fig. 2).

### Dual-tDCS leads to significant changes in TMS metrics in stroke survivors and in controls

We then wanted to explore the effects of dual-tDCS on our TMS metrics. We first wished to investigate whether stroke survivors and controls responded differently to dual-tDCS. We therefore ran two mixed-design 2 × 2 × 3 ANOVAs, with group (patients, controls) as the between-subjects factor, stimulation (real, sham) and time (0, 15, 30 min) as within-subjects factors and normalized TMS measures (MEP, iSP, SICI) as the dependent variable for each hemisphere separately. There was no significant group difference between stroke survivors and controls in response to tDCS in either M1_Anode_ or M1_Cathode_ in any of the TMS metrics (all p’s > 0.1).

**Fig. 3:**
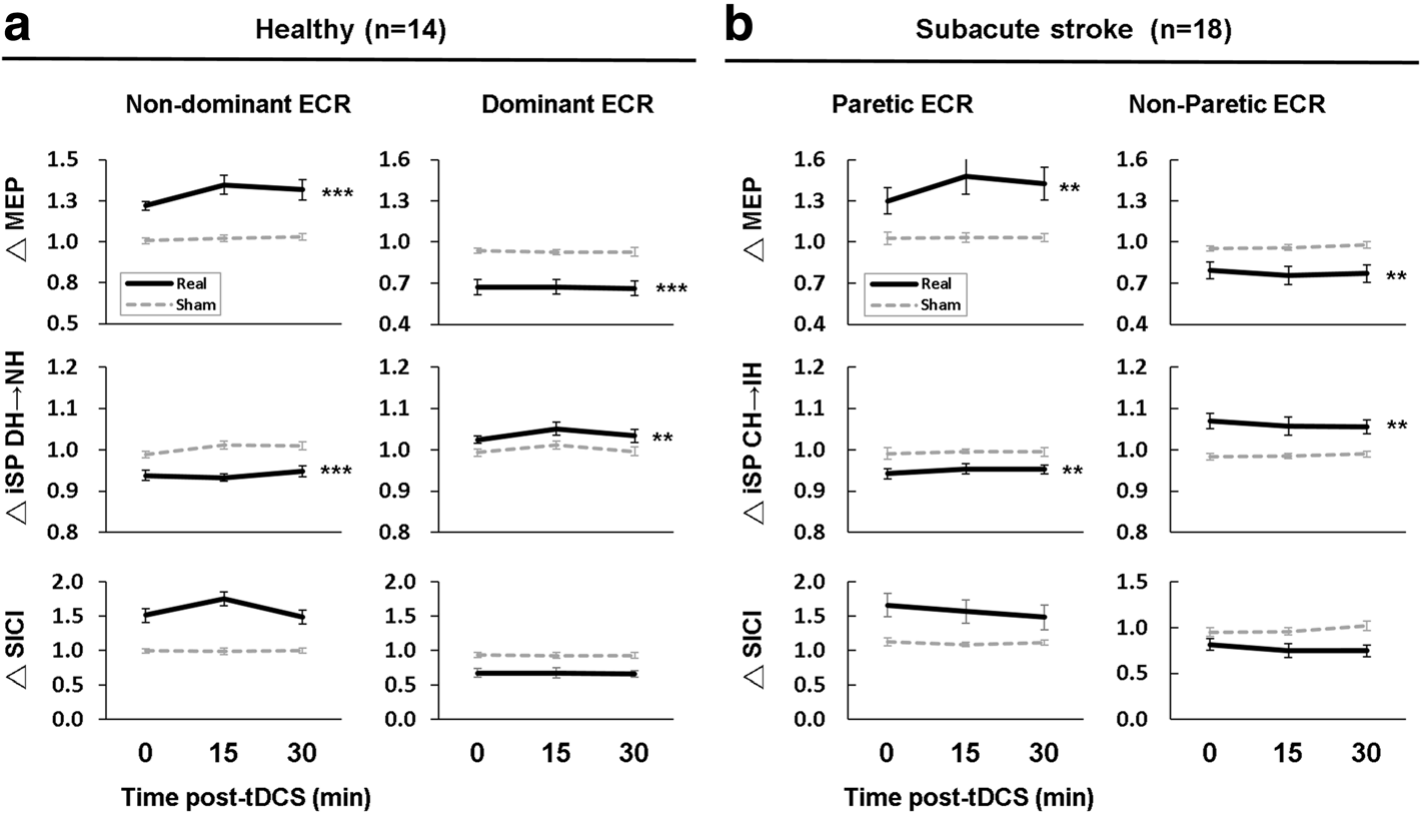
The effect of dual-tDCS on TMS measures over time. **a** Transcranial magnetic stimulation (TMS) metrics recorded from extensor carpi radialis (ECR) at 0, 15 and 30 min after real (solid line) and sham (dotted line) dual-transcranial direct current stimulation (tDCS) for healthy controls (*n* = 14) and (**b**) stroke survivors (*n* = 18). ΔMEP denotes normalised motor evoked potential (MEP), i.e. MEP post tDCS / MEP before tDCS. The same applies for ipsilateral silent period (iSP) and short-interval intracortical inhibition (SICI). Mean and standard error across individuals is shown. DH = dominant hemisphere; NH = non-dominant hemisphere; IH = ipsilesional hemisphere; CH = contralesional hemisphere. *p < 0.05, **p < 0.01, ***p < 0.001

We were interested in the effects of dual-tDCS on TMS measures in stroke survivors to understand group-specific features (Fig. 3). In each group, a set of 2 × 3 repeated measures ANOVAs with factors of stimulation (real, sham) and time (0, 15 min, 30 min) were performed for post-tDCS changes of each normalized TMS measure (MEP, iSP, SICI) from M1_Anode_ and M1_Cathode_ separately.

In stroke survivors, there were significant main effects of stimulation [M1_Anode_: F (1,16) = 12.7, *p* = 0.003, η_p_^2^ = 0.44; M1_Cathode_ F (1,17) = 11.23, *p* = 0.004, η_p_^2^ = 0.40], with an increase MEP amplitude in M1_Anode_ and a decrease in MEP amplitude in M1_Cathode_ after real stimulation relative to sham. There were no significant main effects of time or interactions between stimulation and time (see Additional file 1, Table A.2 with full statistics).

In terms of iSP there were significant main effects of stimulation [M1_Anode_: F (1,15) = 13.7, *p* = 0.002, η_p_^2^ = 0.48; M1_Cathode_: F (1,15) = 12.5, p = 0.003, η_p_^2^ = 0.46], reflecting a shorter iSP in M1_Anode_ and a longer iSP in M1_Cathode_ after real stimulation relative to sham. There were no significant main effects of time, and no significant interactions (Table A.2).

There were no main effects or interactions in either M1 in terms of SICI change (all p’s > 0.1, Table A.2). In line with the non-significant effects of group between stroke survivors and controls, the results in controls were highly similar to those in stroke survivors (Fig. 3, Table A.3).

### Stroke survivors exhibit smaller baseline ipsilesional α- and β- ERSs than controls

**Fig. 4:**
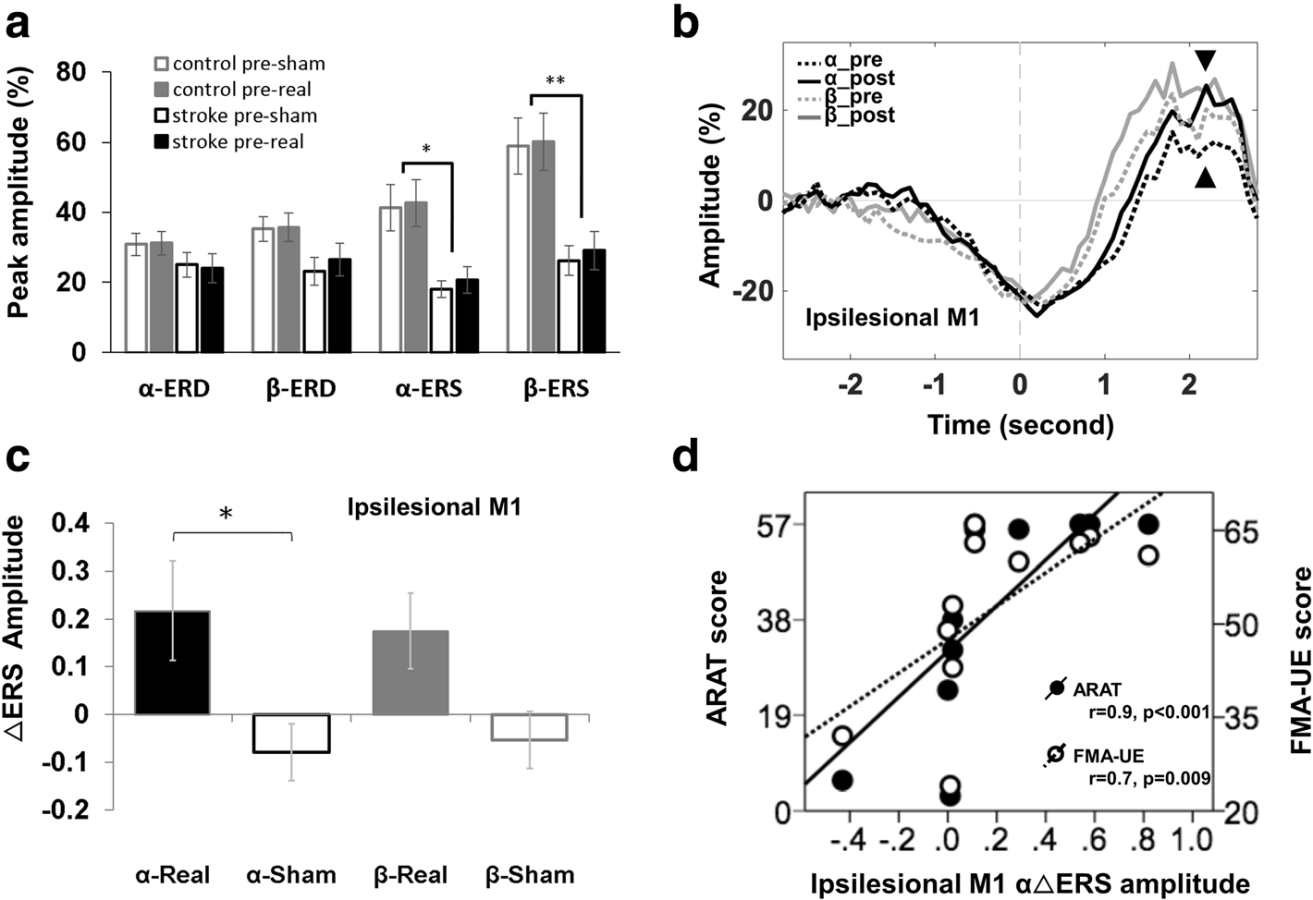
The effect of dual-tDCS in movement-related neural power in stroke survivors (*N* = 11). **a** The baseline peak amplitude of ipsilesional (or right for healthy controls) primary motor cortex (M1) event-related desynchronization (ERD) and event-related synchronization (ERS) in α- and β-frequency band before transcranial direct current stimulation (tDCS) was applied. **b** Time course of movement-related power in the ipsilesional M1 in α (black) and β (gray) frequency band before (dashed) and after (solid) tDCS. The effects of modulation mostly occurred during ERS of α band as indicated by black arrowheads. **c** Same as A, but the average across individuals of the individuals’ strongest ERS deflection is shown. **d** Correlation between stimulation-related change of ipsilesional M1 α-ERS (difference between real and sham tDCS) and motor function (ARAT: closed circles and sloid regression line; FMA-UE: open circles and dashed regression line). * *p* < 0.025, ** *p* < 0.005, α = 0.025

We then wished to see whether the changes in neural excitability as revealed by TMS was reflected in changes in frequency-specific neural activity as measured using MEG. MEG data were acquired from 12 healthy controls and 11 stroke survivors. Initially, we wanted to investigate whether there were any differences in MEG metrics (α-ERD, α-ERS, β-ERD, β-ERS) before tDCS, between stroke survivors and controls (Fig. 4a). Since there were no differences in any baseline MEG metric between the two baseline measures in either group (all p’s > 0.1), the mean of the two baseline measures were used for subsequent analyses. The mixed model ANOVAs and statistics details were summarized in Table A.4. The baseline ERS peak amplitudes in the ipsilesional [right for controls] hemisphere was significantly smaller in stroke survivors than controls in both the α- [U = 27, *p* = 0.015, r = 0.51] and β-frequency bands [U = 17, *p* = 0.002, r = 0.64]. Baseline ERS peak amplitude in the contralesional [left for controls] hemisphere was significantly smaller in stroke survivors than controls in the β-band [U = 20, *p* = 0.005, r = 0.59], but not in the α-band [U = 32, *p* = 0.036, r = 0.44, α = 0.025].

### Dual-tDCS leads to an increase in α-ERS in M1_Anode_ in stroke survivors

Next, we wished to investigate the neural response to dual-tDCS. We first wished to examine whether there was a significant difference between stroke survivors and controls in response to stimulation. We therefore performed a set of 2 × 2x2 mixed-design ANOVAs with group (patients, controls) as a between-subjects factor, stimulation (real, sham) and frequency band (α, β) as within-subjects factors, and normalized MEG measure as dependent variable for M1_Anode_-ERD, M1_Anode_-ERS, M1_Cathode_-ERD and M1_Cathode_-ERS separately. These demonstrated that there were no significant differences between controls and stroke survivors in response to dual-tDCS in either hemisphere (all p’s > 0.1).

We then wanted to explore the group-specific effects of dual-tDCS in stroke survivors alone. Two 2 × 2 × 2 repeated measures ANOVAs with factors of stimulation (real, sham), frequency band (α, β) and time window (ERD, ERS) were performed for M1_Anode_ and M1_Cathode_ separately. In M1_Anode_, there was a significant main effect of stimulation [F (1,10) = 6.04, *p* = 0.034, η_p_^2^ = 0.38], but not of frequency band or time window. There was a significant interaction between stimulation and time window [F (1,11) = 5.44, *p* = 0.042, η_p_^2^ = 0.35]. Therefore, two 2x2 repeated measures ANOVAs with factors of stimulation (real, sham) and frequency band (α, β) were performed for M1_Anode_-ERD and M1_Anode_-ERS separately. No significant differences were observed in M1_Anode_-ERD (all p’s > 0.1). However, there was a significant main effect of stimulation in M1_Anode_-ERS, [F (1,10) = 10.88, *p* = 0.008, η_p_^2^ = 0.52], but no significant main effect of frequency band nor stimulation by frequency band interaction. *Post-hoc* testing revealed that α-ERS in M1_Anode_ significantly increased after stimulation compared with sham (t (10) = 3.19, *p* = 0.01, d = 0.96). There was also a trend of increase in β-ERS in M1_Anode_ after stimulation compared with sham [t (10)=2.42, *p* = 0.036, d = 0.73, α = 0.025; Fig. 4b and c, full statistics in Table A.5).

The equivalent analyses in M1_Cathode_ revealed no significant effects of stimulation (Table A.5). There were no significant effects of stimulation in controls in any MEG metric studied (all p’s > 0.1).

### Dual-tDCS-induced change in α-ERS in ipsilesional M1 correlates with behaviour

This study was performed to understand the neurophysiological underpinnings of tDCS post-stroke. We therefore wished to explore the relationship between the observed stimulation-related increased α-ERS in the ipsilesional M1_Anode_, to address the hypothesis that tDCS-induced changes in neurophysiological measures may relate to functional status. We demonstrated that the stimulation-related change in α-ERS (i.e. change with real stimulation compared to sham) was positively correlated with concurrent paretic upper limb motor scores (r = 0.904, *p* < 0.001 for ARAT; r = 0.744, *p* = 0.009 for FMA-UE, Fig. 4d), such that stroke survivors with better function had larger stimulation-related α-ERS change in ipsilesional M1. No significant correlations were demonstrated between tDCS-induced change in any TMS measure and tDCS-induced change in any other MEG metrics (all p’s > 0.1).

### Responsiveness to dual-tDCS was predicted by interhemispheric inhibition imbalance, as reflected by iSP ratio

There was no statistically difference in response rate between stroke survivors and controls. In M1_Anode_ 93% of controls were “high” responders and 7% “medium”, 61% of stroke survivors were “high” responders and 28% “medium” [χ^2^(2) = 4.40, *p* = 0.07]. In M1_Cathode_ the rates were 86 and 7% in controls and 67 and 5% in stroke survivors [χ^2^(2) = 2.89, *p* = 0.24]. 79% of controls and 44% of stroke survivors were high responders in both hemispheres [χ^2^(1) = 3.80, *p* = 0.075]. Only 3 stroke survivors were “low” responders in both hemispheres, all of whom had relatively severe hand paresis (cases 3, 8, 15).

**Fig. 5:**
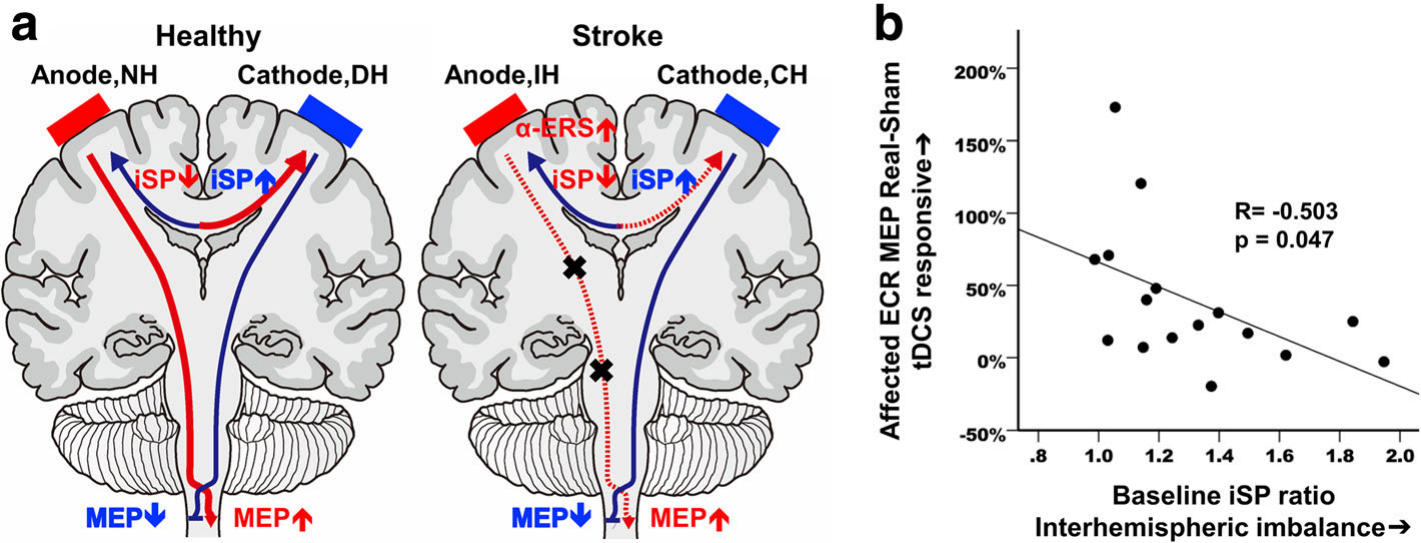
The modulation effects of dual-tDCS and its responsiveness prediction model. **a** The summarized modulation effects of dual-transcranial direct current stimulation (tDCS) in anodal and cathodal polarities in healthy controls and stroke survivors. **b** The dual-tDCS responsiveness in paretic hand of subacute stroke survivors could be predicted from baseline ipsilateral silent period (iSP) ratio. In linear regression analysis, the baseline iSP ratio, i.e. contralesional / ipsilesional hemispheric iSP before tDCS, could significantly and negatively predict changes of normalized motor evoked potentials (MEP), i.e. MEP post tDCS / MEP before tDCS in real relative to sham stimulation. α-ERS: Alpha band event related synchronization; NH: non-dominant hemisphere; DH: dominant hemisphere; IH: ipsilesional hemisphere; CH: contralesional hemisphere

A summary schematic of the neural changes elicited by dual-tDCS in both stroke survivors and controls is given in Fig. 5a. Given the variability in response to tDCS, we believed it would be important to explore the physiological underpinnings of this variability if possible. We therefore ran a multiple regression in stroke survivors to predict MEP change (change of MEP amplitude 15 min after real tDCS stimulation relative to sham) from six independent variables: age, gender, baseline FMA-UE score, baseline MEP ratio (baseline MEP amplitude of ipsilesional hemisphere (IH) relative to contralesional hemisphere (CH)), baseline iSP ratio (CH → IH baseline iSP / IH → CH baseline iSP), and baseline α-ERS ratio (baseline M1 ERS peak amplitude of IH relative to CH). After eliminating the least significant variables (*p* = 0.59 for age, *p* = 0.99 for gender, *p* = 0.57 for FMA-UE, *p* = 0.52 for baseline MEPs ratio, and *p* = 0.41 for baseline α-ERS ratio), the greatest explanatory power for the responsiveness to tDCS was achieved with the iSP ratio (R^2^ = -0.253, *p* = 0.047). In this study, stroke survivors showed significantly higher iSP ratio than controls [Stroke survivors 1.22 (0.99–1.95) [Median (Range)]; Controls 1.02 (0.99–1.95); U = 29.0, *p* = 0.001, r = 0.63)]. Greater baseline iSP ratios predicted a smaller response to tDCS (Fig. 5b). Similar analysis applied in healthy controls using the same independent variables (except baseline FMA-UE) revealed no significant predictors.

## Discussion

To our knowledge, this is the first study to demonstrate the changes of motor cortical excitability, transcallosal inhibition and neural oscillations in subacute stroke survivors after M1-M1 dual-tDCS stimulation. We demonstrated that dual-tDCS led to an increase in cortical excitability and a decrease in transcallosal inhibition, as reflected by a shorter iSP, in the anode-targeted, ipsilesional M1. We also demonstrated a decrease in excitability and an increase in transcallosal inhibition, in the cathode-targeted, contralesional M1. The interhemispheric balance in iSP ratio predicted an individual’s response to subsequent dual-tDCS. In addition, we demonstrated an increase in movement-related ERS in the α-band. The increase in α-ERS was correlated with the stroke survivors’ concurrent ARAT and FMA-UE.

### Increased interhemispheric inhibition to ipsilesional M1 in stroke survivors was significantly modulated by dual-tDCS and predicted response to stimulation

In line with previous literature [53–55], we demonstrated a significant decrease in excitability in the ipsilesional hemisphere and a relatively excessive transcallosal inhibition from the contralesional to ipsilesional hemisphere at baseline, compared with controls, as evidenced by a decreased MEP amplitude and an increased iSP duration. The iSP ratio reflects the degree of asymmetry interhemispheric inhibition, with higher ratio representing more transcallosal inhibition from contralesional [left in controls] M1 toward ipsilesional [right in controls] M1. In addition, this interhemispheric inhibition asymmetry predicted response to dual-tDCS: the greater the inhibition from the contralesional towards ipsilesional M1, the less the ipsilesional M1 excitability could be modulated. This finding is consistent with the hypothesis that excessive transcallosal inhibition may further inhibit ipsilesional hemisphere excitability [4] and, therefore, decrease neuromodulatory response. This hypothesis is supported by animal studies, highlights the role of post-stroke inhibition in motor recovery [56]. The iSP ratio was higher at baseline in stroke survivors than controls and predicted response to tDCS in stroke survivors, but not in controls. It may be, therefore, that while the underlying physiological processes this metric represents is modulated by tDCS in health and disease, it has a functionally important role in representing interhemispheric imbalance. However, we may have to consider the individual difference when applying brain stimulation based on the interhermispheric imbalance hypothesis [7, 38].

The ipsilesional SICI has been suggested to decrease within 3 months post-stroke compared to contralesional and healthy control SICI in a recent meta-analysis by McDonnell et.al [54]. However, we found comparable ipsilesional SICI between subacute (2–4 weeks post-stroke) patients and healthy controls (Fig. 2d). The inconsistency may arise from different stroke severity (our patients had milder paresis than those with significant SICI decrease) and other stroke profiles (our patients were more homogenous as subacute and subcortical infarction) [54]. Further studies are required to determine ipsilesional SICI changes in subacute stroke. The iSP reflects interhemispheric inhibition mediated by γ-Aminobutyric acid (GABA)-A and GABA-B receptors [53, 57], while SICI reflects intra-cortical inhibition mediated by GABA-A activity. Our stroke survivors showed more interhemispheric inhibition than controls, and this was modulated to a greater extent in stroke survivors than controls by dual-tDCS. Both GABA-A and GABA-B circuits have been reported to be modulated by unilateral tDCS [58–60], but none has been explored in dual-tDCS studies.

### Initially decreased ipsilesional M1 α-ERS in stroke survivors increased after tDCS and correlated with motor function

The way in which dual-tDCS modulates sensorimotor rhythms in subacute stroke survivors has not yet been investigated from the literature. Here, we demonstrated that stroke survivors had decreased ERS in both the α- and β-bands in ipsilesional M1 compared with controls. In addition, tDCS-induced change in α-ERS was positively correlated with motor function (FMA-UE and ARAT). Since ERS is thought to represent post-movement cortical inhibition or fine movement control [61, 62] and increased during stroke recovery [63], tDCS may promote stroke motor recovery by modulating ipsilesional M1 circuit interneuronal activity following movement to obtain better motor control.

Some previous studies have demonstrated an increase in α-ERD amplitude during motor imagery after dual- or unilateral- anodal tDCS in chronic stroke [34, 64] and healthy adults [33, 65]. It is not clear why ERD changes were not observed here. One possibility may be derived from the differences between motor imagery and motor execution tasks (we adopted the latter only), which include different cortical excitability states, intracortical circuits and task complexity [66, 67].

### Stroke survivors and controls showed similar high responsiveness to dual-tDCS

In our stroke survivors, the response rate and overall magnitude of the modulation effects in MEP were similar to controls (Fig. 3). Few tDCS studies have addressed responsiveness, but our cohort were much more responsive than those in a previous study using unilateral anodal tDCS [68]. One previous dual-tDCS study [27] in six subacute stroke survivors without healthy controls demonstrated no MEP changes. Further studies are required to compare responsiveness across different tDCS montages and stages of post-stroke motor recovery.

There are some limitations to this study. First, we enrolled a homogenous group of stroke survivors for more consistent dual-tDCS effects in subacute stroke, which may limit the interpretation of our results in other stroke groups. Second, while we observed robust neurophysiological effects of a single session of tDCS on upper limbs, we did not seek to quantify its behavioural effects or concurrent influences on lower limbs. It is therefore difficult to fully describe the potential clinical significance of our findings, though as discussed above, changes in pathophysiological markers may be seen as robust proxy markers of function.

## Conclusions

Our study demonstrated that dual-tDCS modulates neurophysiological markers in post-stroke survivors. These findings add to our understanding of the effects of tDCS in this understudied population. Our results, particularly that inter-hemispheric inhibition asymmetry may influence the effects of dual-tDCS, are important for the design of future clinical studies, suggesting that baseline electrophysiological measures should be therefore considered in translational applications of tDCS in stroke recovery.

## Supplementary information

**Additional file 1.** Table A.1, Table A.2, Table A.3, Table A.4, Table A.5. Full statistic values of analysis of variance.

## Data Availability

The datasets used and analysed during the current study are available from the corresponding author on reasonable request.

## Abbreviations

aMT: Active motor threshold
ANOVA: Analysis of variance
ARAT: Action research arm test
ECR: Extensor carpi radialis
EEG: Electroencephalography
EOG: Electrooculography
ERD: Event-related desynchronization
ERS: Event-related synchronization
FMA-UE: Fugl-Meyer assessment-upper extremity scale
ISI: Inter-stimulus interval
iSP: Ipsilateral silent period
M1: Primary motor cortex
MEG: Magnetoencephalography
MEP: Motor evoked potentials
MRC: Medical Research Council scale of muscle strength
mRS: Modified Rankin Scale
NIHSS: National Institutes of Health Stroke Scale
rMT: Resting motor threshold
SICI: Short-interval intracortical inhibition
tDCS: Transcranial direct current stimulation
TMS: Transcranial magnetic stimulation
