# Supplementary material for: Neurophysiological signatures of hand motor response to dual-transcranial direct current stimulation in subacute stroke: a TMS and MEG study
Source: J Neuroeng Rehabil. 2020 Jun 11;17:72. doi: 10.1186/s12984-020-00706-1 (PMC7291576; doi:10.1186/s12984-020-00706-1)
Supplement: Supplementary file 1 — Additional file 1. Table A.1, Table A.2, Table A.3, Table A.4, Table A.5. Full statistic values of analysis of variance. [file 12984_2020_706_MOESM1_ESM.docx]

**Additional file 1**

**Table A.1 Statistics of baseline TMS metrics (real v.s. sham dual-tDCS)**

| **Healthy controls** | **Left M1** | | **Right M1** | | **Repeated measures ANOVA** |
| --- | --- | --- | --- | --- | --- |
|  | **Sham** | **Real** | **Sham** | **Real** |  |
| rMT (%) | 37±1.4 | 38±1.4 | 39±1.6 | 37±1.4 | F(1,13)=0.601, p=0.452, η_p_^2^=0.35 |
| aMT (%) | 30±1.4 | 30±1.7 | 32±1.6 | 30±2.2 | F(1,13)=0.561, p=0.467, η_p_^2^=0.04 |
| MEP (uV) | 776±97 | 730±108 | 861±125 | 952±250 | F(1,13)=0.053 , p=0.821, η_p_^2^=0.004 |
| iSP (ms) | 57±1.3 | 58±1.2 | 57±1.7 | 57±1.9 | F(1,13)=0.591, p=0.456, η_p_^2^=0.043 |
| SICI (ratio) | 0.27±0.03 | 0.26±0.03 | 0.26±0.02 | 0.29±0.02 | F(1,13)=0.239, p=0.633, η_p_^2^=0.018 |
| **Stroke survivors** | **Ipsilesional M1** | | **Contralesional M1** | | **Repeated measures ANOVA** |
|  | **Sham** | **Real** | **Sham** | **Real** |  |
| rMT (%) | 51±4.9 | 52±4.8 | 39±1.4 | 39±1.6 | F(1,17)=1.824 , p=0.195, η_p_^2^=0.097 |
| aMT (%) | 40±2.9 | 40±2.7 | 33±1.4 | 33±1.4 | F(1,17)=0.000 , p=1.000, η_p_^2^=0.000 |
| MEP (uV) | 298±63 | 266±49 | 927±144 | 967±120 | F(1,17)=0.014 , p=0.906, η_p_^2^=0.001 |
| iSP (ms) | 79±3.2 | 83±3.6 | 64±2.2 | 61±2.8 | F(1,15)=0.675 , p=0.424, η_p_^2^=0.043 |
| SICI (ratio) | 0.27±0.04 | 0.26±0.03 | 0.33±0.02 | 0.34±0.04 | F(1,17)=0.007 , p=0.933, η_p_^2^=0.000 |

TMS: transcranial magnetic stimulation; M1: primary motor cortex; rMT: resting motor threshold; aMT: active motor threshold, MEP: motor evoked potential; iSP: ipsilateral silent period; SICI: short interval intracortical inhibition

**Table A.2 Statistics of TMS metrics in stroke survivors (real v.s. sham dual-tDCS)**

| **MEP** | M1_Anode_ | M1_Cathode_ |
| --- | --- | --- |
| [Stimulation]^1^ | F(1,16)=12.7, **p=0.003,** η_p_^2^=0.44 | F(1,17)=11.2, **p=0.004**, η_p_^2^=0.40 |
| [Time] | F(2,32)=3.52, p=0.06, η_p_^2^=0.18 | F(2,32)=0.31, p=0.73, η_p_^2^=0.18 |
| [Stimulation x Time] | F(2,32)=2.17, p=0.13, η_p_^2^=0.12 | F(2,34)=0.75, p=0.48, η_p_^2^=0.04 |
| **iSP** | M1_Anode_ | M1_Cathode_ |
| [Stimulation]^2^ | F(1,15)=13.7, **p=0.002**, η_p_^2^=0.48 | F(1,15)=12.5, **p=0.003**, η_p_^2^=0.46 |
| [Time] | F(2,30)=0.49, p=0.62, η_p_^2^=0.03 | F(2,30)=0.40, p=0.67, η_p_^2^=0.03 |
| [Stimulation x Time] | F(2,30)=0.09, p=0.91, η_p_^2^=0.006 | F(2,30)=0.75, p=0.48, η_p_^2^=0.05 |
| **SICI** | M1_Anode_ | M1_Cathode_ |
| [Stimulation] | F(1,14)=1.87, p=0.19, η_p_^2^=0.12 | F(1,14)=1.52, p=0.24, η_p_^2^=0.10 |
| [Time] | F(2,28)=0.40, p=0.67, η_p_^2^=0.03 | F(2,28)=1.25, p=0.30, η_p_^2^=0.08 |
| [Stimulation x Time] | F(2,28)=0.28, p=0.76, η_p_^2^=0.02 | F(2,28)=0.94, p=0.40, η_p_^2^=0.06 |

TMS: transcranial magnetic stimulation; M1: primary motor cortex; rMT: resting motor threshold; aMT: active motor threshold, MEP: motor evoked potential; iSP: ipsilateral silent period; SICI: short interval intracortical inhibition

**Table A.3 Statistics of TMS metrics in healthy controls (real v.s. sham dual-tDCS)**

| **MEP** | M1_Anode_ | M1_Cathode_ |
| --- | --- | --- |
| [Stimulation]^1^ | F (1, 13)=58.4, **p<0.001,** η_p_^2^=0.82 | F (1, 13)=44.9, **p<0.001**, η_p_^2^=0.79 |
| [Time] | F (2, 26)=3.04, p=0.065, η_p_^2^=0.19 | F (2, 26)=1.34, p=0.28, η_p_^2^=0.10 |
| [Stimulation x Time] | F (2, 26)=2.1, p=0.14, η_p_^2^=0.14 | F (2, 24)=0.22, p=0.80, η_p_^2^=0.02 |
| **iSP** | M1_Anode_ | M1_Cathode_ |
| [Stimulation]^2^ | F (1, 13)=24.1, **p<0.001**, η_p_^2^=0.65 | F(1, 13)=5.94, **p=0.03**, η_p_^2^=0.31 |
| [Time]^3^ | F(2, 26)=3.93, **p=0.03**, η_p_^2^=0.23 | F(2, 26)=5.68, **p=0.009**, η_p_^2^=0.30 |
| [Stimulation x Time] | F (2, 26)=2.02, p=0.15, η_p_^2^=0.13 | F (2, 26)=0.14, p=0.87, η_p_^2^=0.01 |
| **SICI** | M1_Anode_ | M1_Cathode_ |
| [Stimulation] | F(1, 12)=1.41, p=0.26, η_p_^2^=0.11 | F(1, 12)=1.07, p=0.32, η_p_^2^=0.08 |
| [Time] | F(2, 24) =1.29, p=0.29, η_p_^2^=0.10 | F(2, 24)=2.31, p=0.12, η_p_^2^=0.16 |
| [Stimulation x Time] | F(2, 24)=1.42, p=0.26, η_p_^2^=0.11 | F(2, 24)=1.30, p=0.29, η_p_^2^= 0.10 |

TMS: transcranial magnetic stimulation; M1: primary motor cortex; rMT: resting motor threshold; aMT: active motor threshold, MEP: motor evoked potential; iSP: ipsilateral silent period; SICI: short interval intracortical inhibition

^1^The MEP amplitude increased in M1_Anode_ and decreased in M1_Cathode_ after real stimulation relative to sham.

^2^The iSP was shorter in M1_Anode_ and longer in M1_Cathode_ after real stimulation relative to sham.

^3^*post-hoc* tests with Bonferroni correction showed a lower iSP change immediately after stimulation when compared to the iSP change 30 minutes post-stimulation in M1_Anode_ (p=0.019); a higher iSP change 15 minutes post-stimulation when compared to the iSP change immediately after stimulation in M1_Cathode_ (p=0.007).

**Table A.4 Statistics of baseline ERD and ERS between stroke survivors and healthy controls**

|  | ERD | ERS |
| --- | --- | --- |
| [Group] | F(1,21)=0.04, p=0.85, ηp2=0.002 | F(1, 21)=11.2, **p=0.003**, η_p_^2^=0.35 |
| We ran two 2x2x2 mixed-design ANOVAs with group (patients, controls) as the between-subject factor, frequency band (α, β) and hemisphere (ipsilesional [right for controls], contralesional [left for controls]) as within subject factors, and MEG metric peak amplitude (ERD or ERS) as dependent variable. There was a significant difference between controls and stroke survivors in ERS [F(1,21)=11.2, p=0.003, ηp2=0.35], but not in ERD [F (1,21)=0.04, p=0.85, ηp2=0.002]. | | |
|  | Ipsilesional [right for controls] ERS | Contralesional [left for controls] ERS |
| [Group] | F(1,21)=11.8, **p=0.002**, η_p_^2^=0.36 | F(1,21)=8.18, **p=0.009**, η_p_^2^=0.28 |
| Therefore, we ran two 2x2 mixed-design ANOVA with a between-subject factor of group (patients, controls), a within-subject factor of band (α, β), and baseline ERS peak amplitude as the dependent variable for either hemisphere. There were significant effects of group in both the ipsilesional [right for controls] hemisphere ERS [F(1,21)=11.8, p=0.002, ηp2=0.36] and contralesional [left for controls] hemisphere ERS [F(1,21)=8.18, p=0.009, ηp2=0.28].  *Post-hoc* testing revealed that the baseline ERS peak amplitudes in the ipsilesional [right for controls] hemisphere was significantly smaller in stroke survivors than controls in both the α- [U=27, p=0.015, r=0.51] and β-frequency bands [U=17, p=0.002, r=0.64]. Baseline ERS peak amplitude in the contralesional [left for controls] hemisphere was significantly smaller in stroke survivors than controls in the β-band [U=20, p=0.005, r=0.59], but not in the α-band [U=32, p=0.036, r=0.44, α=0.025]. | | |

**Table A.5 Statistics of tDCS effects on ERD and ERS in stroke survivors**

|  | M1_Anode_ | M1_Cathode_ |
| --- | --- | --- |
| [Stimulation] | F(1,10)=6.04, **p=0.034**, ηp2=0.38 | F (1, 10)=4.42, p=0.062, η_p_^2^=0.31 |
| [Frequency band] | F(1,10)=0.83, p=0.38, ηp2=0.08 | F (1, 10)=0.94, p=0.36, η_p_^2^=0.09 |
| [Time window] | F(1,10)=1.87, p=0.20, ηp2=0.16 | F (1,10)=1.10, p=0.33, η_p_^2^=0.10 |
| [Stimulation x Time window] | F(1,11)=5.44, **p=0.042**, ηp2=0.35 | - |
| We wanted to explore the group-specific effects of dual-tDCS in stroke survivors alone. Two 2x2x2 repeated measures ANOVAs with factors of stimulation (real, sham), frequency band (α, β) and time window (ERD, ERS) were performed for M1_Anode_ and M1_Cathode_ separately. In M1_Anode_, there was a significant main effect of stimulation, but not of frequency band or time window. There was a significant interaction between stimulation and time window. In M1_Cathode_ revealed no significant effects of stimulation. | | |
|  | M1_Anode_ ERD | M1_Anode_ ERS |
| [Stimulation] | F(1,10)=0.02, p=0.88, η_p_^2^=0.002 | F(1,10)=10.88, **p=0.008**, η_p_^2^=0.52 |
| [Frequency band] | F(1, 10)=0.78, p=0.40, η_p_^2^=0.07 | F(1,10)=0.023, p=0.883, η_p_^2^=0.002 |
| [Stimulation x Frequency band] | - | F(1,10)=0.48, p=0.50, η_p_^2^=0.046 |
| Therefore, two 2x2 repeated measures ANOVAs with factors of stimulation (real, sham) and frequency band (α, β) were performed for M1_Anode_ ERD and M1_Anode_ ERS separately. No significant differences were observed in M1_Anode_ ERD. However, there was a significant main effect of stimulation in M1_Anode_ ERS, but no significant main effect of frequency band nor stimulation by frequency band interaction. Then, *post-hoc* tests ran as reported in the main manuscript. | | |

M1: primary motor cortex; ERD: event-related desynchronization; ERS: event-related synchronization; ANOVA: analysis of variance.
